# New alleles for chlorophyll content and stay-green traits revealed by a genome wide association study in rice (*Oryza sativa*)

**DOI:** 10.1038/s41598-019-39280-5

**Published:** 2019-02-22

**Authors:** Yan Zhao, Chenggen Qiang, Xueqiang Wang, Yanfa Chen, Jinqiang Deng, Conghui Jiang, Xingming Sun, Haiyang Chen, Jin Li, Weilan Piao, Xiaoyang Zhu, Zhanying Zhang, Hongliang Zhang, Zichao Li, Jinjie Li

**Affiliations:** 1 Key Laboratory of Crop Heterosis and Utilization of the Ministry of Education, and Beijing Key Laboratory of Crop Genetic Improvement, China Agricultural University, Beijing, 100193 China; 2 Department of Plant Science, Plant Genomics and Breeding Institute, and Research Institute of Agriculture and Life Sciences, Seoul National University, Seoul, 08826 Republic of Korea

## Abstract

Higher chlorophyll content (CC) and strong stay-green (SG) traits are conducive for improvement of photosynthetic efficiency in plants. Exploration of natural elite alleles for CC and SG, and highly resolved gene haplotypes are beneficial to rational design of breeding for high-photosynthetic efficiency. Phenotypic analysis of 368 rice accessions showed no significant correlation between CC and SG, and higher CC and stronger SG in *japonica* than in *indica*. Genome-wide association studies of six indices for CC and SG identified a large number of association signals, among which 14 were identified as pleiotropic regions for CC and SG. Twenty-five known genes and pleiotropic candidate gene *OsSG*1 accounted for natural variation in CC and SG. Further analysis indicated that 20 large-effect, non-synonymous SNPs within six known genes around GWAS signals and three SNPs in the promoter of *OsSG*1 could be functional causing significant phenotypic differences between alleles. Superior haplotypes were identified based on these potentially functional SNPs. Population analyses of 368 cultivated accessions and 446 wild accessions based on SNPs within genes for CC and SG suggested that these genes had been subjected to strong positive selection in *japonica* in the process of spreading from its subtropical origin to the North China temperate zone. Our studies point to important genes that account for natural variation and provide superior haplotypes of possible functional SNPs that will be beneficial in breeding for high-photosynthetic efficiency in rice.

## Introduction

Chlorophylls is essential for photosynthesis and mainly functions in light energy harvesting and as a transfer center^1^. Chlorophyll content (CC) is closely related to crop yield as a consequence of photosynthetic efficiency, but degradation of chlorophyll during maturation limits yield potential because of leaf senescence^2^. Breeding crop genotypes with prolonged active photosynthetic duration is a practical approach to increase yield^3^, but requires strong stay-green (SG) phenotypes. Rice is a stable food source that feeds more than half of the world population, and insight into the genetic basis of CC and SG could provide valuable information for breeding.

Chlorophyll biosynthesis and degradation have been studied biochemically and genetically in various organisms^4–6^. Hundreds of genes related to CC have been reported in rice. Among them, a large number of genes were detected using rice mutants exhibiting yellow-green (chlorina) or albinic leaves, such as *YGL8*, *OsDVR*, *OsValRS2*, *YGL138(t)*, *OsNUS1*, *v2*, *PAPST1*, *GIC*, *RNRL2*, *RNRL1*, *RNRS1* and *CHR72*^7–17^. Some genes affecting chlorophyll content were identified using SG mutants, such as *NYC1*, *NOL* and *SGR*^18,19^. These studies identified functional genes controlling CC and SG, but natural variation in those genes was not revealed. Therefore, detailed knowledge of the natural variation in genes underlying CC and SG is required in order to identify or design superior cultivars with highly efficient photosynthetic capacity.

Rich genetic diversity and clear subspecies and population differentiation are established characteristics of Asian cultivated rice^20–24^. These natural variations underlie adaptability to different light and temperature conditions that range from tropical to temperate zones, and also lay a genetic basis for breeding specifically adapted varieties with high and stable yields. Compared with conventional linkage mapping in biparental populations, genome-wide association studies (GWAS) explore a wider range of natural variation and enable identification of numerous SNPs associated with targeted traits. Using diverse rice accessions, the genetic architecture of natural variation in rice CC was investigated through GWAS, and genes *Ghd7* and *NAL* were found to be associated with CC^25^. Given that molecular breeding relies on precise genetic dissection of agronomic traits and high-resolution chromosome haplotypes^26^, further identification of elite alleles underlying CC and SG will be beneficial in gaining insights into the molecular basis of variation in CC and SG and in breeding photosynthetically efficient varieties.

In the present study, we identified ontological gene categories and overview of 152 known genes controlling CC and SG. The genetic architecture of natural variation in CC and SG was studied through GWAS using 368 cultivated Asian rice accessions. Twenty-five known genes and candidate gene *OsSG1* accounted for natural variation in CC and SG. We scanned for possible functional non-synonymous SNPs within these genes, and observed diverse effects of the major haplotypes. In addition, we investigated the signatures of natural selection on genes underlying variation in CC and SG within and between *indica* and *japonica* subpopulations. The results provide insight into how domestication has affected CC and SG genes as well as information that may be useful for future molecular applications of these genes in breeding for high photosynthetic efficiency.

## Materials and Methods

### Materials and sequencing data

Three hundred and sixty-eight rice accessions from 32 countries were used as materials for identification of CC and SG genes. The sequence data of all accessions were obtained from the 3000 Rice Genome Project (3KRGP)^24,27,28^. For phylogenetic analysis, we added 446 wild rice accessions, having publicly available sequencing data from a previous report^20^.

### Phenotyping

All 368 rice accessions were used in phenotyping CC and SG. Field experiments were performed at the China Agricultural University Shangzhuang Experimental Station in Beijing in the summer of 2014. Two replicates were grown in each of two fields and each accession was transplanted 30 days after sowing in three row plots with 20 cm between plants and 26 cm between rows. Three central plants from the middle row of each plot were used to assess CC and SG. We measured the CC in the flag leaf, and second and third upper leaves of two tillers of each plant by a SPAD (soil-plant analysis development) meter (SPAD-502 Plus, Konica-Minolta, Japan) at heading and 30 days after heading. Average SPAD values across the two replicates were used for analysis.

We adopted six indices to evaluate the CC and SG of all materials. These included SPAD of the flag leaf at heading (SFH), total SPAD for the three upper leaves at heading (TSH), absolute difference value of SPADs of the flag leaf at heading and 30 days post heading (ADSF), relative difference value of SPAD of the flag leaf at heading and 30 days post heading (RDSF), cumulative SPAD of the flag leaf at heading and 30 days post heading (CSF), and total cumulative SPAD for the three upper leaves at heading and 30 days post heading (TCS). The formulae of ADSF, RDSF, CSF and TCS were: ADSF = SPAD of the flag leaf at heading − SPAD of the flag leaf at 30 days post heading, RDSF = ADSF/SPAD of the flag leaf at heading, CSF = SPAD of the flag leaf at heading + SPAD of the flag leaf at 30 days post heading, and TCS = total SPAD for the three upper leaves at heading + total SPAD for the three upper leaves at 30 days post heading. Among these indices, SFH and TSH were used as CC indices, and ADSF and RDSF indicated the difference and degradation rate of CC at two growth stages. We applied the two indices to assess the SG of each accession. We also considered CSF and TCS as indices to evaluate ability including CC and SG, which to a certain extent, reflect the accumulation of chlorophyll (ACC) during the heading and 30 days post heading stages.

### Population genetic analysis and GWAS

More than 3.3 million SNPs with minor allele frequencies (MAF) >0.05 and missing rates <0.5 were used in population genetic analysis and GWAS. Principal component (PC) and kinship analyses were performed using GAPIT^29^ to evaluate population structure and relative kinship of the 368 rice accessions. The first three PCs were used to construct a PC matrix. To control spurious associations, we performed GWAS on 6 indices for CC, SG and ACC using the compressed mixed linear model (CMLM) with PC and kinship matrices, that account for population structure and identify the optimal group kinship matrix^30^. A significance threshold was calculated using the formula “-log_10_(1/the effective number of independent SNPs)” as described previously^31^, and effective numbers of independent SNPs were determined by PLINK to be 144605, 172233 and 95342 in the full population, and *indica* and *japonica* subpopulations, respectively^32^. The suggestive *P* values were 6.9 × 10^−6^, 5.8 × 10^−6^ and 1.0 × 10^−5^, respectively. Finally, the threshold was set at −log(*P*) = 5 to identify significant association signals. Due to different genome-wide linkage disequilibrium (LD) decay rates in *indica* and *japonica* at 123 kb and 167 kb^33^, adjacent significant SNP with distances less than 170 kb were merged into single association signals. The SNP with the minimum *P* value in a signal region was considered to be the lead SNP. In order to identify candidate genes in the signal region, LD heatmaps surrounding peaks in the GWAS were constructed using the R package “LD heatmap”^34^.

### GO and KEGG pathway enrichment analysis

A cytoscape plug-in ClueGO v2.3.5 was used to analyse GO and pathway enrichment^35^. According to the default parameters, a two-sided hypergeometric test and Bonferroni stay-down correction were used to identify enriched GO terms and pathways. Significant enrichment was detected with a corrected *P* value of <0.05.

### Non-synonymous SNPs and haplotype analysis

Based on information on coding sequence (CDS) coordinates and the transcript from MSU RGAP 7, we separated non-synonymous SNPs from all SNPs across the 368 accessions using an in-house Perl script. Differences in phenotypic values between alleles of each non-synonymous SNP were examined by Student’s t-tests. Sequence alignment of each gene was performed using non-synonymous SNPs associated with CC or SG, and differences in phenotypic values among haplotypes of each gene were calculated by one-way ANOVA or Student’s t-tests. Duncan’s multiple range tests were conducted to make comparisons if the results of the one-way ANOVA were significant (*P* < 0.05).

### Phylogenetic relationships and identification of selective signals

A phylogenetic tree for all 368 cultivated and 446 wild accessions was constructed using the neighbor-joining method in TASSEL 5 and MEGA 5^36,37^. Nucleotide diversity (π)^38^ and Tajima’s *D*^39^ were calculated using an in-house Perl script.

## Results

### Population structure and phenotypic characterization of CC, SG and ACC of cultivated rice

PC and kinship analysis showed that the sampled material could be divided into two subpopulations comprising 199 *indica* and 169 *japonica* accessions (Fig. S1). Large variations were observed in the whole population among CC indices SFH and TSH, SG indices ADSF and RDSF, and ACC indices CSF and TCS (Fig. S2). High correlations were detected between paired CC, SG and ACC indices with correlation coefficients of 0.943, 0.968 and 0.912, respectively (Table S1). High correlation coefficients (>0.7) were also detected between the CC and ACC indices, whereas low negative correlations were detected between the SG and ACC indices. A low correlation coefficient (<0.4) between the CC and SG indices (Table S1) suggested that there were distinct genetic architectural differences between CC and SG, and that a higher CC index did not imply enhancement of SG.

Taking into account the large genetic differences between the subspecies^20^, we compared CC and SG between *indica* and *japonica*. Two CC indices for *indica* were significantly lower than those for *japonica* (Table S2). Clear differences were detected between *indica* and *japonica* for two SG indices (Table S2). Phenotypic variation in ADSF and RDSF for *indica* ranged from 0 to 35 and from 0 to 1, whereas phenotypic variation in ADSF and RDSF for *japonica* ranged from 0 to 20 and from 1 to 0.4, respectively (Fig. S2). Moreover, higher ACC was detected in *japonica* than in *indica* (Fig. S2). These results suggested that *japonica* rice has higher CC, stronger SG and higher ACC than *indica*.

### Fourteen loci for CC and SG were detected by GWAS

**Figure 1:**
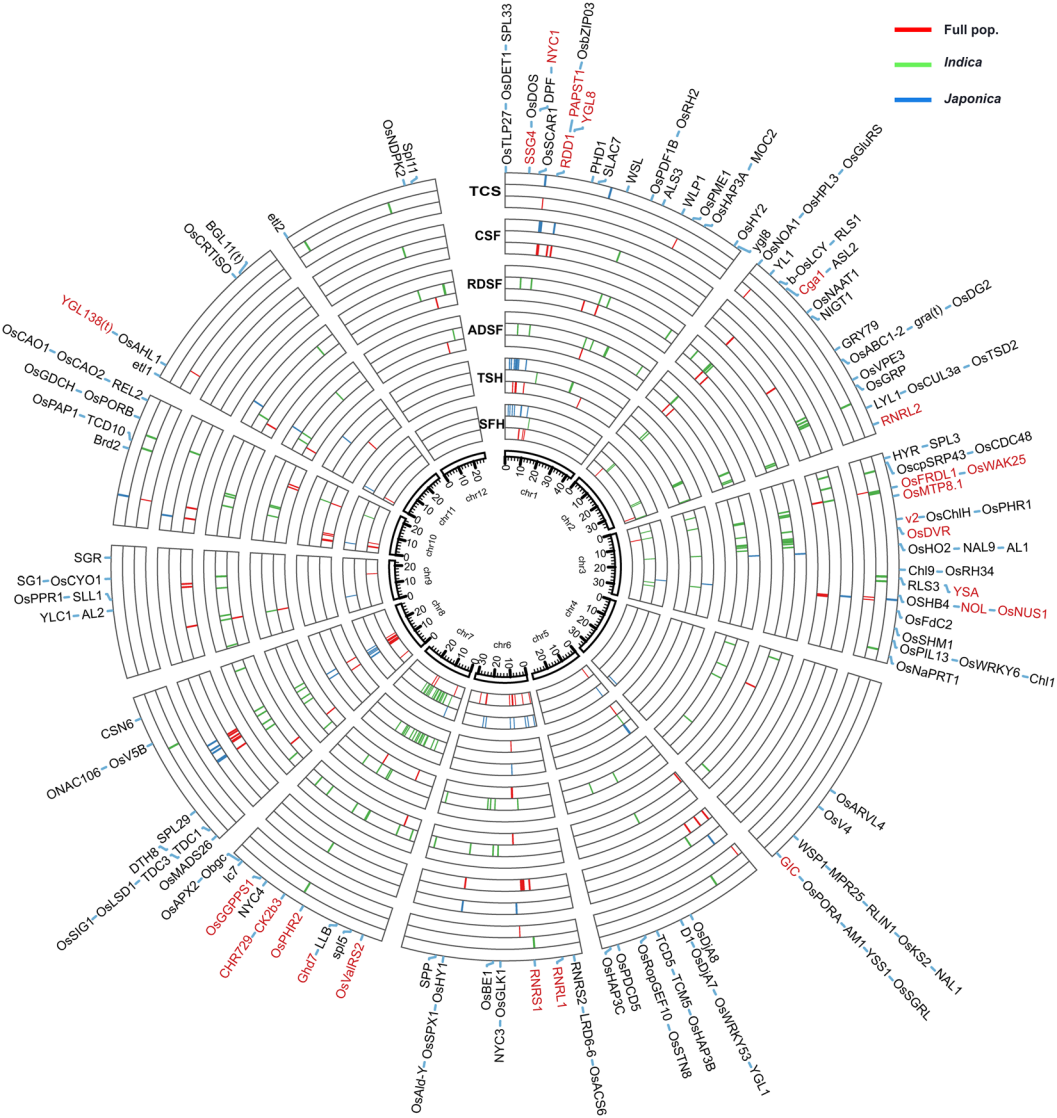
Circos map of all association signals for six indices of chlorophyll content and stay-green in the full population, *indica* and *japonica*. One hundred and fifty-two known genes are labeled at the outermost layer where red color represents known genes around GWAS signals in our association analysis.

A GWAS was performed to identify associations of SNPs for CC, SG and ACC in the full population, and in the *indica* and *japonica* subpopulations under CMLM (Materials and methods). Thirty-five, 15, 13, 12, 28 and 10 significant signals were obtained for SFH, TSH, ADSF, RDSF, CSF and TCS, respectively, in the full population (Figs 1 and S3; Table S3). In *indica*, 30, 30, 48, 53, 12 and 15 significant signals were identified for SFH, TSH, ADSF, RDSF, CSF and TCS, respectively (Figs 1 and S4; Table S3), whereas there were 27, 15, 2, 2, 13 and 4 significant signals for the corresponding indices in the *japonica* subpopulation (Figs 1 and S5; Table S3). The differences in the number of significant signals between the subspecies were due to larger phenotypic variation in *indica* than that in *japonica*.

There were 28 common lead SNPs for the separate CC indices in GWAS among the three subpopulations, and most significant signals showed overlapping with multiple significant SNPs clustered in regions of less than 170 kb (Fig. 1; Table S3). Six common lead SNPs for SFH were identified using the full population and *japonica* subpopulation (Fig. 1; Table S3). However, no common lead SNP for CC was detected between the *indica* and *japonica* subpopulations (Fig. 1; Table S3). These results indicated an obvious genetic heterogeneity between *indica* and *japonica*.

More genetic heterogeneity in SG was detected between *indica* and *japonica* by comparing GWAS results for the two subpopulations. Not only was there no common lead SNP in *indica* and *japonica*, but only two signals for SG were found in *japonica* (Fig. 1; Table S3). Considering less significant association signals and narrower phenotypic variation of SG in *japonica*, we suggest that strong SG and low genetic diversity of related genes may be important characteristics of *japonica*.

ACC is a complex trait that includes CC and SG. By comparison with GWAS results for CC, fifteen common lead SNPs were associated with CSF and SFH, and three common lead SNPs were associated with TCS and TSH (Fig. 1; Table S3). Thus several genes were responsible for ACC and CC.

To further examine associations for CC and SG, we compared significant lead SNPs detected in the three populations for CC and SG. Fourteen pleiotropic association regions for CC and SG were identified (Fig. 1; Table 1), and among them, eight were also identified for ACC. These results suggested that there were several pleiotropic genes for CC and SG.

**Table 1:** Fourteen regions associated with chlorophyll content and stay-green phenotype.

Chr	Significant signals	Trait and population
1	3538227, 3608401, 3691807, 3818693, 3856943	TSH_*Jap*., ADSF_*Ind*., RDSF_*Ind*., TSH_Full, TSH_*Jap*.
1*	7017944, 7020023, 7020293, 7022643	(SFH_Full and TSH_*Jap*.), TSH_Full, ADSF_*Ind*., SFH_*Jap*.
2	34757389, 34912592, 34919467	SFH_Full, RDSF_*Ind*., ADSF_*Ind*.
6	9642009, 9712936	ADSF_*Ind*., SFH_Full
6*	10972220, 11013054, 11013746, 11092024	SFH_Full, (SFH_*Jap*., TSH_*Jap*.), TSH_Full, ADSF_Full
6*	21488969, 21551988	SFH_*Jap*., ADSF_*Ind*.
7	4565962, 4732672, 4797040	RDSF_*Ind*., (SFH_*Ind*., TSH_*Ind*.), SFH_*Jap*.
7	12518256, 12671000, 12741399, 12843936	RDSF_*Ind*., ADSF_*Ind*., RDSF_*Ind*., (SFH_*Ind*. and TSH_*Ind*.)
7*	15821905, 15932240, 16073851, 16135435	TSH_*Ind*., SFH_*Ind*., SFH_Full, (ADSF_Full, ADSF_*Ind*. and RDSF_*Ind*)
8*	10928247, 11065511	(ADSF_*Ind*. and RDSF_*Ind*.), SFH_*Jap*.
8	12403163, 12441193, 12447246	SFH_Full, SFH_*Jap*., (ADSF_*Ind*. and RDSF_*Ind*.)
8*	14488622, 14600128, 14670703, 14733920	SFH_*Jap*., TSH_Full, (ADSF_*Ind*. and RDSF_*Ind*.), TSH_*Jap*.
9*	6729748, 6762082, 6769841, 6770962	SFH_*Jap*., RDSF_*Ind*., ADSF_Full, (ADSF_*Ind*. and RDSF_Full)
10*	16428207, 16505539, 16648136	TSH_Full, (SFH_Full and TSH_Full), ADSF_Full

^*^Regions significantly associated with CC, SG and ACC.

Adjacent significant signals with distances less than 170 kb were merged as a single QTL.

### Natural variation in genes responsible for CC and SG

Comprehensive analysis of known genes is conducive to exploration and utilization of loci responsible for natural variation in CC and SG. One hundred and fifty two known genes associated with CC (leaf color) or SG in rice were selected from the China Rice Data Center (http://www.ricedata.cn/) database and more recent reports^25,40–42^. The gene ontology (GO) categories significantly enriched in this protein group were located in chloroplasts (Fig. S6), and mainly involved ‘porphyrin-containing compound metabolism’ (Fig. S7) by adjusting the activity of various reductases (Fig. S8). We analyzed the metabolic processes associated with the 152 genes. ‘Porphyrin and chlorophyll metabolism’ was the only significantly enriched metabolic pathway and included 16 known genes (Fig. S9). The combined analysis of GO and pathway of these genes showed that CC and SG were controlled by a complex network, with a large number of proteins for CC and SG being located in chloroplasts and involved in metabolism of porphyrin-containing compounds.

To identify large-effect genes affecting CC and SG in natural rice populations, we performed further comparisons between the 152 known genes and GWAS data obtained in this study. Genes *SSG4* (LOC_Os01g08420), *NYC1* (LOC_Os01g12710), *RDD1* (LOC_Os01g15900), *PAPST1* (LOC_Os01g16040), *YGL8* (LOC_Os01g17170), *OsWAK25* (LOC_Os03g12470), *OsMTP8*.*1* (LOC_Os03g12530), *NOL* (LOC_Os03g45194), *OsNUS1* (LOC_Os03g45400), *GIC* (LOC_Os04g57920), *RNRL1* (LOC_Os06g07210), *RNRS1* (LOC_Os06g14620), *OsValRS2* (LOC_Os07g06940), *Ghd7* (LOC_Os07g15770), *OsPHR2* (LOC_Os07g25710), *CK2β3* (LOC_Os07g31280) and *CHR729* (LOC_Os07g31450) were located in the association regions for two CC indices, indicating that these genes could contain important loci involved in natural variation of CC; *NYC1* (LOC_Os01g12710), *Cga1* (LOC_Os02g12790), *RNRL2* (LOC6_Os02g56100), *OsMTP8*.*1* (LOC_Os03g12530), *v2* (LOC_Os03g20460), *OsDVR* (LOC_Os03g22780), *OsGGPPS1* (LOC_Os07g39270) and *YGL138(t)* (LOC_Os11g05552) were located in association regions for two SG indices, suggesting that they could be related to natural variation in SG; *NYC1* (LOC_Os01g12710), *RDD1* (LOC_Os01g15900), *PAPST1* (LOC_Os01g16040), *YGL8* (LOC_Os01g17170), *OsFRDL1* (LOC_Os03g11734), *OsWAK25* (LOC_Os03g12470), *OsMTP8*.*1* (LOC_Os03g12530), *YSA* (LOC_Os03g40020), *NOL* (LOC_Os03g45194), *OsNUS1* (LOC_Os03g45400) and *RNRS1* (LOC_Os06g14620) were in association regions for two ACC indices, implying that these genes could be involved in natural variation of ACC (Fig. 1; Table S3). Genes *NYC1* and *OsMTP8*.*1* encoding chloroplast-localized proteins were in association regions for CC, SG and ACC (Table S3). Additionally, seven genes, *RDD1*, *PAPST1*, *YGL8*, *OsWAK25*, *NOL*, *OsNUS1* and *RNRS1* were in association regions for CC and ACC (Table S3). Thus 25 known genes around GWAS signals probably have roles in natural variation of CC or SG, especially the first nine genes mentioned above.

### Elite alleles in six cloned genes for CC and SG

**Figure 2:**
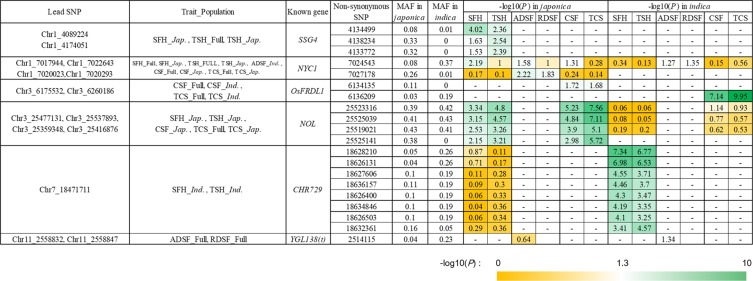
Non-synonymous SNPs within known genes around GWAS signals in GWAS.

Further study was made to identify alleles of known genes for CC and SG. Eight hundred and eleven non-synonymous SNPs were detected within the above 152 genes, including 306 SNPs in the full association panel with MAF > 0.05. For the subpopulations, there were 605 non-synonymous SNPs, including 185 with MAF > 0.05 in *japonica*, and 529 non-synonymous SNPs in *indica* including 232 with MAF > 0.05. Considering the complexity of population structure and genetic background, we performed a statistical analysis of each subpopulation by Student’s t-tests. Eight, 7, 2, 1, 6 and 5 non-synonymous SNPs in *japonica* were significantly (*P* < 0.05) associated with SFH, TSH, ADSF, RDSF, CSF and TCS, respectively. Eight, 8, 1, 1, 1 and 1 non-synonymous SNPs showed significant associations with SFH, TSH, ADSF, RDSF, CSF and TCS in *indica*, respectively (Fig. 2).

**Figure 3:**
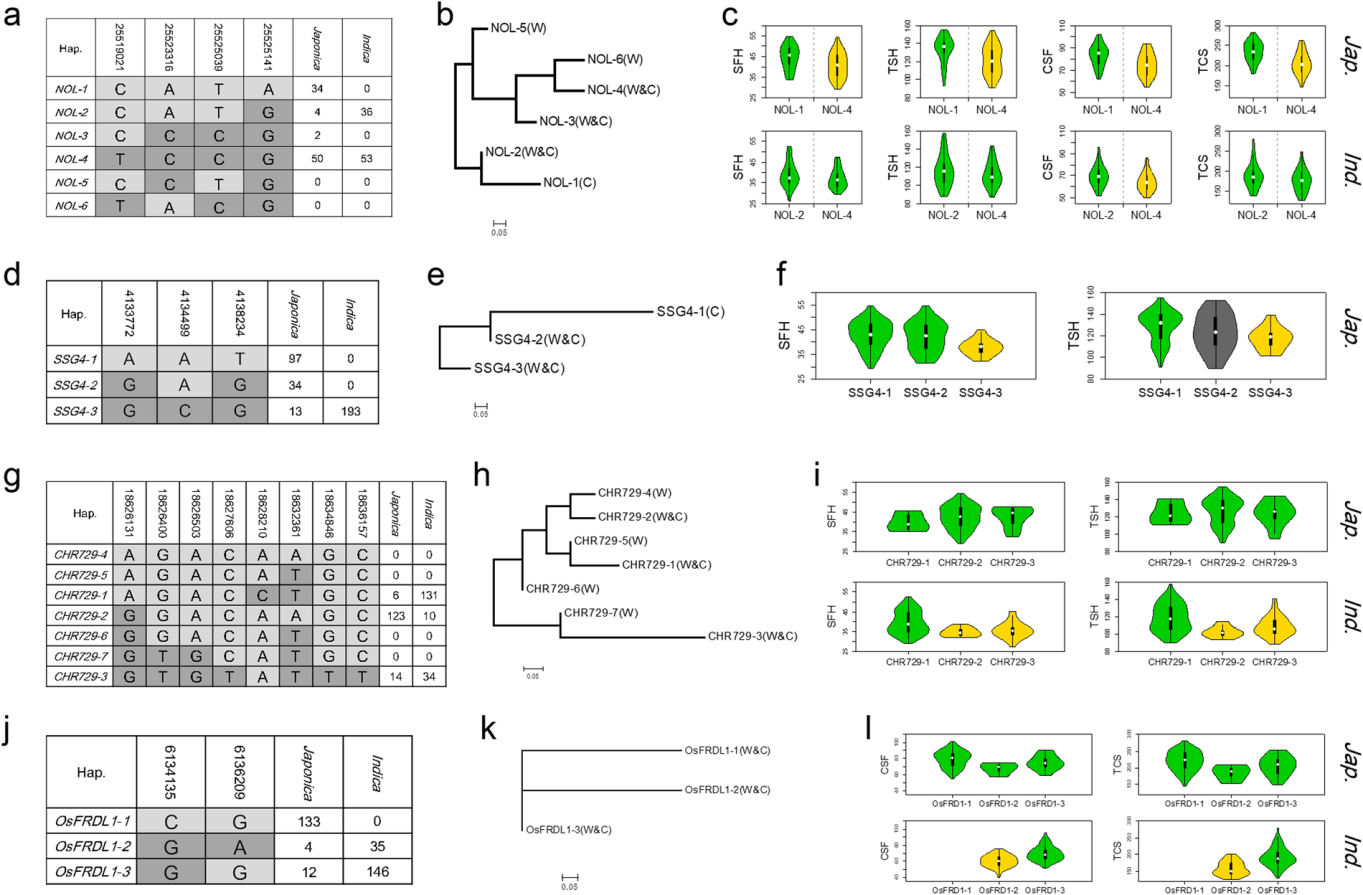
Haplotype analysis of *NOL*, *SSG4*, *CHR729* and *OsFRDL1*. Gene structures of (**a**) *NOL*, (**d**) *SSG4*, (**g**) *CHR729* and (**j**) *OsFRDL1*. Phylogenetic trees of (**b**) *NOL*, (**e**) *SSG4*, (**h**) *CHR729* and (**k**) *OsFRDL1*. W & C indicate detection in wild and cultivated rice. Comparisons of chlorophyll content indices among (**c**) *NOL*, (**f**) *SSG4*, (**i**) *CHR729* and (**l**) *OsFRDL1* genotypes in *japonica* and *indica*. Green violins show significantly higher values of chlorophyll metabolism than yellow violins.

There were four non-synonymous SNPs (Chr3_ 25519021, Chr3_ 25523316, Chr3_ 25525039 and Chr3_ 25525141) within the *NOL* gene, which encodes a chloroplast-localized short-chain dehydrogenase/reductase (SDR) with three transmembrane domains, and mutation in which produced an SG phenotype^18,43^. These SNPs showed significant associations with four indices for CC and ACC (SFH_*Jap*., TSH_*Jap*., CSF_*Jap*., TCS_Full and TCS_*Jap*.) (Fig. 2). Allele C at Chr3_ 25519021, allele A at Chr3_ 25523316, allele T at Chr3_ 25525039, and allele A at Chr3_25525141 represented higher CC and more ACC in *japonica* (Figs 2 and S10). Six haplotypes, named *NOL-1* to *NOL-6*, were identified based on the four non-synonymous SNPs in wild and cultivated rice (Fig. 3a). *NOL-1* and *NOL-2* were present in *japonica* and *indica* accessions, respectively, and both showed large genetic distances from other haplotypes (Fig. 3b). *NOL-4* was predominant and shared across *japonica*, *indica* and wild rice. There were highly significant differences between *NOL-1* and *NOL-4* among SFH, TSH, CSF and TCS in *japonica* with -log(*P*) values of 2.35, 3.15, 3.36 and 5.34, respectively (Fig. 3c). In *indica*, there were clear differences in SFH, TSH, CSF and TCS between *NOL-2* and *NOL-4* (Fig. 3c). Accessions with the *NOL-1* genotype had higher CC and ACC than accessions having other haplotypes.

Three non-synonymous SNPs (Chr1_4133772, Chr1_4134499 and Chr1_4138234) in known genes around GWAS signals for CC were detected within *SSG4*, mutation of which affected the size of chloroplasts and amyloplasts and produced a variegated phenotype^44^. These SNPs showed significant associations with two CC indices in *japonica* (SFH_*Jap*., TSH_Full and TSH_*Jap*.) (Figs 2 and S11). Three haplotypes were present in cultivated and wild rice (Fig. 3d,e). Haplotype *SSG4-1* was prevalent only in the *japonica* population, *SSG4-2* was mostly present in *japonica* and wild rice, and *SSG4-3* was detected in *japonica*, *indica* and wild rice. Highly significant differences were observed in SFH and TSH between *SSG4-1* and *SSG4-3* in *japonica* by one-way ANOVA (*P* < 0.01) (Fig. 3f).

Eight non-synonymous SNPs (Chr7_18626131, Chr7_18626400, Chr7_18626503, Chr7_18627606, Chr7_18628210, Chr7_18632361, Chr7_18634846 and Chr7_18636157) were detected within *CHR729*, mutation of which caused a number of morphological and growth defects, including reduced CC^45^. The eight non-synonymous SNPs showed significant associations with 2 CC indices in *indica* (SFH_*Ind*. and TSH_*Ind*.) (Figs 2 and S12). Three haplotypes, *CHR729-1* to *CHR729-3*, were detected in cultivated and wild rice, and the other four occurred only in wild rice (Fig. 3g,h). SFH and TSH of *CHR729-1*, prevalent in *indica*, were significantly higher than in the other two haplotypes (*P* < 0.01) (Fig. 3i).

For ACC, two non-synonymous SNPs (Chr3_ 6134135 and Chr3_6136209) were identified within *OsFRDL1*. Knockout of this gene resulted in leaf chlorosis^46^. Both SNPs were significantly associated with two ACC indices in *indica* (CSF_Full, CSF_*ind*., TCS_Full and TCS_*Ind*.) (Figs 2 and S13). Three haplotypes were present in cultivated and wild rice, and there was a large genetic difference between *japonica* and *indica* (Fig. 3j,k). Haplotype *OsFRDL1-1* was present in *japonica*, and *OsFRDL1-2* and *OsFRDL1-3* predominated in *indica*. Significant differences in CSF and TCS were detected between *OsFRDL1-2* and *OsFRDL1-3* with −log(*P*) values of 7.08 and 9.12 in *indica* by Student’s t-tests (Fig. 3l).

Two (Chr1_7024543 and Chr1_7027178) and one (Chr11_2514115) non-synonymous SNPs within *NYC1* and *YGL138(t)* showed significant associations with CC or SG, respectively (Fig. 2). The *NYC1* mutant is a stay-green mutant in which chlorophyll degradation during senescence is impaired^18^, and the *YGL138(t)* mutant exhibits a distinct yellow-green leaf phenotype throughout development^47^. There were two haplotypes within *NYC* and *YGL138(t)* in each subpopulation due to rare non-synonymous SNPs and obvious differentiation of *indica* and *japonica*. In conclusion, the 20 non-synonymous SNPs could be possible functional SNPs within six known genes responsible for natural variation in CC and SG; natural elite alleles/haplotypes were identified for the six known genes with larger effects on CC and SG.

### Variation in *OsSG1*, a new locus for CC, SG and ACC

**Figure 4:**
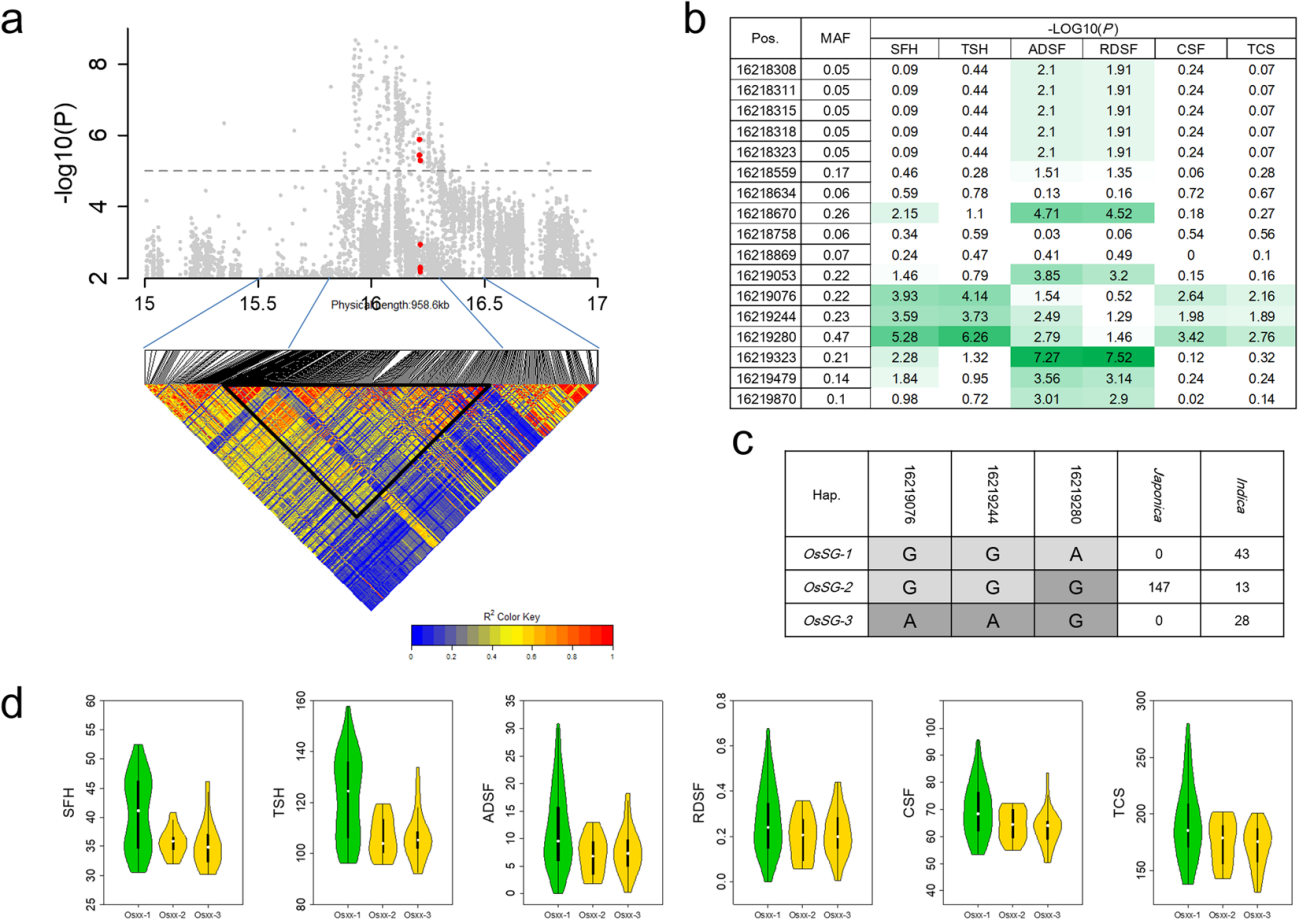
Exploration of *OsSG1* for chlorophyll content and stay-green on chromosome 7. (**a**) Local Manhattan plot (top) and LD heatmap (bottom) surrounding the lead SNP for SFH on chromosome 7. Red dots show all SNPs within *OsSG1*. (**b**) Comparison of six indices for chlorophyll content and stay-green between alleles of SNPs in *indica* using Student’s t-test. (**c**) Gene structures of *OsSG1*. (**d**) Comparison of six indices for chlorophyll content and stay-green among haplotypes of *OsSG1* in *indica* using one-way ANOVA. Green violins show significantly higher phenotypic values than yellow violins (*P* < 0.05).

We found an association region at 15-17 Mb on chromosome 7, in which lead SNPs at Chr7_15932240, Chr7_15821905, Chr7_16135435, Chr7_16135435, Chr7_16023159 and Chr7_15821905 in *indica* were associated with SFH, TSH, ADSF, RDSF, CSF and TCS with −log(*P*) values of 8.66, 7.60, 6.57, 5.14, 5.82 and 5.89, respectively (Figs 4a and S14). Lead SNPs at Chr7_16073851 and Chr7_16135435 in the full population were associated with SFH and ADSF with −log(*P*) values of 5.99 and 5.76, respectively (Fig. S14). By using pairwise LD correlations (*r*^2^ > 0.6)^48,49^, we estimated a candidate region from 15.8 Mb to 16.3 Mb (Fig. 4a). High *r*^2^ values were detected among the four lead SNPs in *indica* (Fig. S15). The results suggested that there could be a single pleiotropic gene regulating CC, SG and ACC within the LD block.

Stable expression of 20 of 70 annotated genes within the candidate region was detected in rice leaves (Table S4). By GO analysis of the 20 genes, we found candidate gene LOC_Os07g27790 encoding a protein with glutamate-cysteine ligase activity that participated in glutathione biosynthesis. This gene was predicted to be located in plastids. Metabolic pathway analysis using the KEGG system suggested that LOC_Os07g27790 could be involved in glutathione metabolism, together with known genes *RNRS2*, *RNRS1*, *OsAPX2* and *RNRL1* for chlorophyll content. According to these analyses, we suggest that LOC_Os07g27790, named as *OsSG1*, is an important candidate gene controlling multiple chlorophyll-related traits, including CC, SG and ACC.

To explore possible functional sequences within *OsSG1* based on re-sequencing data, we investigated associations between six indices and non-synonymous SNPs as well as SNPs located in the 5′ flanking sequence (≤2 Kb upstream of the open reading frame) of *OsSG1*. Three non-synonymous SNPs were identified but their MAFs were lower than 0.05 in *indica* (Fig. S16). Considering that these associated signals were detected in the *indica* subpopulation, we postulated that the three non-synonymous SNPs could not be the cause of the variation affecting CC and SG. Eighteen SNPs with MAF > 0.05 were detected in the promoter of *OsSG1* in *indica*; 14 of them showed significant associations with at least one of 6 indices for CC, SG and ACC (Fig. 4b); three adjacent SNPs in set of 14 (Chr7_16219076, Chr7_16219244, Chr7_16219280) were associated with almost all indices for CC, SG and ACC (Fig. 4b). The haplotypes of *OsSG1* were assembled using re-sequencing data of the three SNPs; three haplotypes were detected in *indica*, and all *japonica* accessions carried *OsSG1-2* (Fig. 4c). Clear differences were observed in all six indices in *indica* between *OsSG1-1* and *OsSG1-2* and between *OsSG1-1* and *OsSG1-3* by one-way ANVOA (Fig. 4d). Varieties carrying *OsSG1-1* showed higher CC (SFH = 40.6 and TSH = 122) and ACC (CSF = 69.7 and TCS = 191), but weaker stay-green capacity (ADSF = 11.6 and RDSF = 0.27) than *indica* varieties carrying *OsSG1-2* or *OsSG1-3*. The results suggested the sequences in *OsSG1* for maintaining protein function were highly conserved, and that phenotypic differences between the three haplotypes could be caused by the differences in expression level in *indica*.

### Strong positive selection on genes related to CC and SG in *japonica*

**Figure 5:**
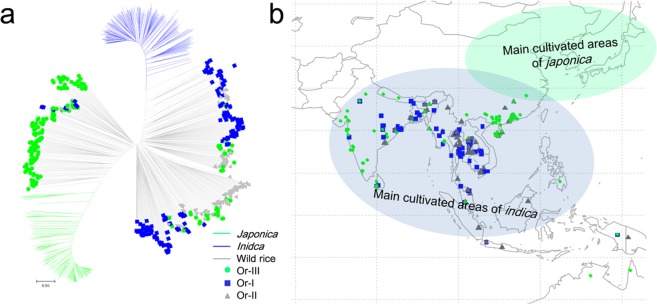
Phylogenetic relationships and geographical distribution of cultivated and wild rice accessions. (**a**) Phylogenetic tree of 368 cultivated and 446 wild accessions using SNPs within genes related to chlorophyll content and stay-green. (**b**) Main areas of cultivated rice and geographical distribution of wild rice.

In order to investigate the domestication history of genes related to CC and SG in *indica* and *japonica*, we made a phylogenetic analysis and signature identification of selection using 368 cultivated and 446 wild rice accessions. According to the phylogenetic tree calculated from SNPs in 152 known genes and *OsSG1*, there was a distinct differentiation between *japonica* and *indica* (Fig. 5a). *Japonica* accessions were close to the Or-III (*japonica*-like wild rice) group from southern China, and *indica* accessions were close to Or-I (*indica*-like wild rice) (Fig. 5a). Thus the SNPs in *japonica* could be inherited from Or-III, whereas those in *indica* were from Or-I. Selective signal scans were performed within the CC and SG genes using the ratio of genetic diversity in wild rice to that in *japonica* and *indica* (π_W_/π_J_ and π_W_/π_I_), respectively (Table S5). Twenty-eight and 55 known genes showed high selective signals in *indica* (π_W_/π_I_ > 3) and *japonica* (π_W_/π_J_ > 3), respectively. After considering the values of Tajima’s *D* (Tajima’s *D* < −2) of these genes in their respective subpopulations, we found that nine genes had been strongly positively selected in *indica*, whereas 43 genes were strongly selected in *japonica* (Table 2). By comparing the geographical areas of distribution of cultivated and wild rice we found that *indica* rice and wild rice were mainly distributed in low latitudes with short days and high light intensity, whereas *japonica* was far from its ancestral progenitor (Or-III), and distributed in areas with long days and low light intensity (Fig. 5b). We therefore suggest that genes controlling CC and SG in *japonica* rice were positively selected in the process of spreading from a subtropical origin to the temperate zone of North China.

**Table 2:** Summary of 43 and 9 genes that had undergone positive selection in *japonica* and *indica*, respectively.

Chr.	Gene	π_W_/π_I_	π_W_/π_J_	Tajima’s *D*		
				*Indica*	*Japonica*	Wild rice
1	*MOC2*	11.7	3.3	−2.4	−1.6	−1.2
2	*OsNAAT1*	4.6	1.8	−2.1	−1.8	−1.1
5	*TCD5*	16.1	1.1	−2.7	0.4	−1.3
9	*SLL1*	7.4	1.7	−2.6	−1.7	−1.2
2	*NIGT1*	14.7	6	−2.2	−2.2	−0.2
3	*OsCDC48*	6.7	3.9	−2	−2.7	−1.6
5	*OsHAP3B*	3.6	3.5	−2.1	−2.3	−1.7
7	*Obgc*	39.6	27.6	−2.3	−2.6	−1.2
7	*spl5*	6.4	4.5	−2.2	−2.2	−1.1
1	*OsTLP27*	1.1	3.5	1.5	−2.2	0.2
1	*PHD1*	0.7	3.7	0.7	−2.4	−1.7
1	*SLAC7*	1.6	5.7	−1.2	−2.5	0.2
1	*OsHAP3A*	1.4	3.7	0	−2.2	−1.2
1	*OsSCAR1*	1.3	3.4	0.4	−2	−0.5
1	*RDD1*	0.7	3.7	2.7	−2.3	−0.2
1	*PAPST1*	2.7	14.2	2.4	−2.3	0.9
2	*OsGRP*	1.7	5.1	−1.2	−2.3	−0.6
2	*LYL1*	0.6	8.8	1.6	−2	−0.8
2	*OsCUL3a*	1.3	7.9	−1.2	−2.7	−0.2
2	*OsTSD2*	4.2	5.7	−1.2	−2.3	−1.4
2	*OsHPL3*	0.6	3.5	0.2	−2.5	−0.5
3	*OsPHR1*	5.2	4.6	−1.7	−2.2	0
3	*AL1*	4.1	3	−1.8	−2.3	−0.3
3	*RLS3*	1.1	4.5	0.3	−2.1	−1.4
3	*OSHB4*	1.6	5.7	0.3	−2.4	−0.9
3	*OsSHM1*	2.2	5.6	−0.5	−2.2	−1.2
3	*SPL3*	1.9	3.6	−0.7	−2	−1.2
3	*OsPIL13*	3	6.7	−1.5	−2.4	−0.8
3	*OsNaPRT1*	1.2	5.5	0.4	−2.3	−1.1
4	*RLIN1*	2.5	3.8	−1.9	−2.2	−0.6
4	*GIC*	5.4	5.2	−1.7	−2.3	−1.3
4	*YSS1*	1.6	7.3	−0.6	−2.3	−1.1
4	*OsSGRL*	3.5	6.1	−1.8	−2.5	−0.6
5	*OsRopGEF10*	0.7	3.5	4.7	−2.4	−0.7
6	*LRD6-6*	0.7	3.4	0.3	−2.3	−0.8
6	*SPP*	0.7	4.4	3.5	−2.5	−0.9
8	*CSN6*	1.9	4.2	−0.5	−2.5	−0.5
9	*YLC1*	1.3	3	−0.8	−2.2	1
9	*OsCYO1*	2.1	8.6	−0.2	−2.5	−0.7
9	*SG1*	2.9	4.4	−1.6	−2.4	−0.6
9	*SGR*	1.2	3.6	−0.8	−2.5	−1
10	*TCD10*	0.8	4.5	0	−2.4	1.2
10	*REL2*	1.1	9.1	0.9	−2.7	−1
10	*OsCAO2*	7.9	23.8	−1.6	−2.6	−1.4
10	*OsCAO1*	2.6	8.8	−1.8	−2.7	−1.4
11	*YGL138(t)*	1.1	4.5	−0.5	−2.5	0.7
12	*etl2*	0.8	4.9	−1.2	−2.4	−0.8

## Discussion

### Natural variation in 25 candidate genes has important roles in CC and SG

With development of functional genomics, high throughput genotyping and phenotyping technologies, more than 2,200 genes have been cloned and functionally identified in rice by forward or reverse genetic strategies. Based on those studies, molecular knowledge has been increasingly applied to the breeding of high yielding, superior-quality rice. This is considered to be a powerful strategy to meet the challenges of future crop breeding, particularly in pyramiding multiple complex traits^26^. Despite these research results the practice of breeding by molecular design is still difficult and requires more precise genetic dissection of agronomic traits and precisely identified chromosome haplotypes.

High throughput genotyping and GWAS provide strong support for determining the effect of known functional genes in natural populations and exploration of superior natural variation^25,50^. In this study, we conducted GWAS using a diverse worldwide population of 368 rice accessions, following a comparison of GWAS results and 152 known genes for CC or SG. Twenty-five known genes were around GWAS signals in GWAS, implying that these genes could be involved in genetic variation of CC or SG, and could be used in molecular breeding for high photosynthetic efficiency.

Gene function can be manipulated by alterations in expression level and protein sequence, and polymorphisms causing protein-coding differences are most likely to be important functional SNPs associated with target traits^48^. Based on high-density SNPs from the 3KRGP, we extracted 811 non-synonymous SNPs within known genes for CC or SG. After removing SNPs with MAF < 0.05, 20 non-synonymous SNPs within 6 of 25 genes (*SSG4*, *NYC1*, *OsFRDL1*, *NOL*, *CHR729* and *YGL138(t)*) were associated with at least one of six indices, implying that the 20 SNPs could be real functional SNPs accounting for natural variation in CC or SG. The results of haplotype analysis using the 20 non-synonymous SNPs can provide guidance for pyramiding desirable alleles associated with CC and SG in molecular design of genotypes with high photosynthetic efficiency.

### OsSG1 is a natural variant of CC and SG in *indica*

One important finding in our study was that *OsSG1* might be a major gene accounting for variation in CC, and also control of SG. In GWAS of *indica*, strong signals of six indices around *OsSG1* suggested that there could be a pleiotropic gene regulating CC, SG and ACC in a single LD block. KEGG pathway analysis showed that *OsSG1* was involved in glutathione metabolism, together with four known genes *RNRS1*, *RNRS2*, *OsAPX2* and *RNRL1* for CC or SG. In a previous study^16^, mutants of *RNRS1* and *RNRL1* produced chlorotic leaves in a growth stage-dependent manner under field conditions, and yeast two-hybrid analysis showed that the interacting activities were RNRL1:RNRS1 > RNRL1:rnrs1 > rnrl1:RNRS1 > rnrl1:rnrs1, which correlated with the degree of chlorosis for each genotype^16^. The activity of RNRL1 homolog RNRS2 could supplement RNRS1 activity in chloroplast biogenesis in developing leaves^16^. *OsAPX2* mutants had significantly lower CC than wild-type plants and over-expression increased CC to a level higher than in wild-type plants^51^. Since these genes involved the regulatory mechanism of CC or SG, we suggest that further investigation of the glutathione metabolic network could help in genetic dissection of CC and SG.

### Genes for CC and SG have been subjected to positive selection in *japonica*

Asian cultivated rice is well known for its rich within-species diversity with two major subspecies, *indica* and *japonica*, and further subpopulation differentiation. Previous studies and this study show that CC and SG in *japonica* are significantly higher than in *indica*^25^. However, the pathway of physiological change during domestication of distinct subpopulations remains unclear. Genetic analysis using well-characterized domestication loci indicated that *japonica* and *indica* were close to wild rice subpopulations Or-III and Or-I, respectively. *Japonica* was first domesticated from Or-III in southern China (Fig. 5a). Our phylogenetic tree using SNPs within genes for CC and SG is similar to those of well-characterized domestication loci, implying that higher CC and SG were important domestication traits. Selective signal scans showed that several genes were strongly positively selected in cultivated rice, especially in *japonica* (Table 2). Given the geographical distributions of *japonica*, *indica* and wild rice, higher CC and SG could have enabled *japonica* to adapt to higher latitudes with longer days and lower light intensities (Fig. 5b). However, the phylogenetic tree for each gene for CC and SG showed a distinct domestication pattern (Fig. 3). Among the *NOL* and *SSG4* genes for chlorophyll content, the *NOL-1* and *SSG-1* haplotypes for higher CC levels were detected only in *japonica*, implying that they were new mutations acquired during domestication of *japonica*. All haplotypes of *CHR729* and *OsFRDL1* were detected in wild rice, and *CHR729-2* and *OsFRDL1-1* were prevalent haplotypes in *japonica* whereas *CHR729-1*, *OsFRDL1-2* and *OsFRDL1-3* predominated in *indica*. Our results suggest that during domestication of *japonica*, the planting areas gradually extended from low altitudes to high altitudes along with the changes in light intensity and daylength. During this adaptation new natural mutations for higher CC and SG were preserved, and gradually accumulated along with natural elite variation from wild rice.

## Supplementary information

Supplementary Table S1 Correlation coefficients of six indices in the entire association panel

Supplementary Table S2 Differences between indica and japonica accessions for 6 indices of chlorophyll content and stay-green

Supplementary Table S3 Significant association signal for SFH in full populaiton, indica and japonica

Supplementary Table S4 Expression of 20 genes within candidate region on chromosome 7

Supplementary Table S5 Nucleotide diversity and Tajima's D test for 152 known genes and OsST1.

Supplementary Figures
