# Supplementary material for: New alleles for chlorophyll content and stay-green traits revealed by a genome wide association study in rice (Oryza sativa)
Source: Sci Rep. 2019 Feb 22;9:2541. doi: 10.1038/s41598-019-39280-5 (PMC6384888; doi:10.1038/s41598-019-39280-5)
Supplement: Supplementary file 6 — Supplementary Figures [file 41598_2019_39280_MOESM6_ESM.docx]

**Scientific Reports Supplementary figures**

**New alleles for chlorophyll content and stay-green traits revealed by a genome wide association study in rice (*Oryza sativa*)**

**Running title: Functional SNPs for chlorophyll content and stay-green traits**

Yan Zhao^1, 2#^, Chenggen Qiang^1#^, Xueqiang Wang^1#^, Yanfa Chen^1^, Jinqiang Deng^1^, Conghui Jiang^1^, Xingming Sun^1^, Haiyang Chen^1^, Jin Li^1^, Weilan Piao^3^, Xiaoyang Zhu^1^, Zhanying Zhang^1^, Hongliang Zhang^1^, Zichao Li^1^ & Jinjie Li^1*^

^1^ Key Laboratory of Crop Heterosis and Utilization of the Ministry of Education, and Beijing Key Laboratory of Crop Genetic Improvement, China Agricultural University, Beijing 100193, China.

^2^ State Key Laboratory of Crop Biology, Shandong Key Laboratory of Crop Biology, College of Agronomy, Shandong Agricultural University, Tai'an, Shandong, 271018, PR China

^3^ Department of Plant Science, Plant Genomics and Breeding Institute, and Research Institute of Agriculture and Life Sciences, Seoul National University, Seoul 08826, Republic of Korea.

^#^ These authors contributed equally to this work.

* Correspondence should be addressed to Jinjie Li (Email: [lijinjie@cau.edu.cn](mailto:lijinjie@cau.edu.cn); Tel: +86 010 62734018).

**The following supplementary figures are available for this article:**

**Fig. S1** Population structure of 368 rice accessions used in the study.

**Fig. S2** Histograms of six indices for chlorophyll content (CC), stay-green (SG) and chlorophyll accumulation during heading and 30 days post heading stages (ACC) for the full population (violet), indica (yellow) and japonica (green).

**Fig. S3** Genome-wide association study of six indices for CC, SG and ACC in full population under CMLM.

**Fig. S4** Genome-wide association study of six indices for CC, SG and ACC in *indica* under CMLM.

**Fig. S5** Genome-wide association study of six indices for CC, SG and ACC in *japonica* under CMLM.

**Fig. S6** GO (cellular component) analysis of 152 known genes for CC and SG.

**Fig. S7** GO (molecular function) analysis of 152 known genes for CC and SG.

**Fig. S8** GO (biological process) analysis of 152 known genes for CC and SG.

**Fig. S9** KEGG pathway analysis of 152 known genes for CC and SG.

**Fig. S10** Allele analysis of four non-synonymous SNPs within *NOL* gene.

**Fig. S11** Allele analysis of three non-synonymous SNPs within the *SSG4* gene.

**Fig. S12** Allele analysis of eight non-synonymous SNPs within the *CHR729* gene.

**Fig. S13** Allele analysis of two non-synonymous SNPs within the *OsFRDL1* gene.

**Fig. S14** Genome-wide association signals in the region 15-17 Mb on chromosome 7 for six indices.

**Fig. S15** LD heatmap of four lead SNPs associated with six indices on chromosome 7.

**Fig. S16** Sequence alignment of *OsSG1* using three non-synonymous SNPs.

**Fig. S1 Population structure of 368 rice accessions used in the study. (a)** Principal component and **(b)** kinship analyses of 368 rice accessions, **(c)** PCA eigenvalue.

**Fig. S2 Histograms of six indices for chlorophyll content (CC), stay-green (SG) and chlorophyll accumulation during heading and 30 days post heading stages (ACC) for the full population (violet), *indica* (yellow) and *japonica* (green).**

**Fig. S3 Genome-wide association study of six indices for CC, SG and ACC in full population under CMLM.**

**Fig. S4 Genome-wide association study of six indices for CC, SG and ACC in *indica* under CMLM.**

**Fig. S5 Genome-wide association study of six indices for CC, SG and ACC in *japonica* under CMLM.**


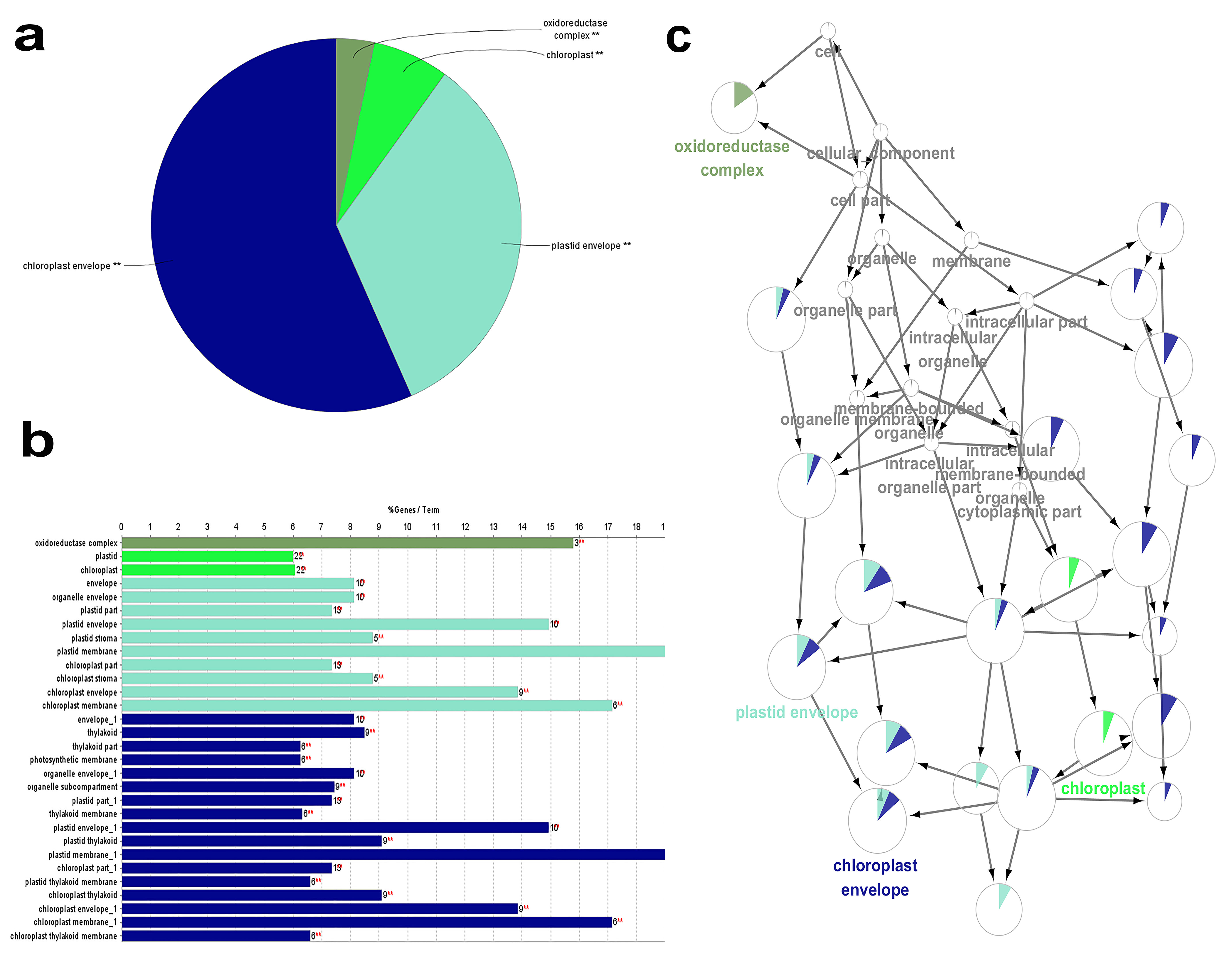


**Fig. S6 GO (cellular component) analysis of 152 known genes for CC and SG. (a)** GO terms specific for known genes. **(b)** Overview chart with functional groups including specific terms for known genes. (**c**) Functionally grouped network with terms as linked nodes based on their kappa score more than 0.3, where only the label of the most significant term per group is shown.


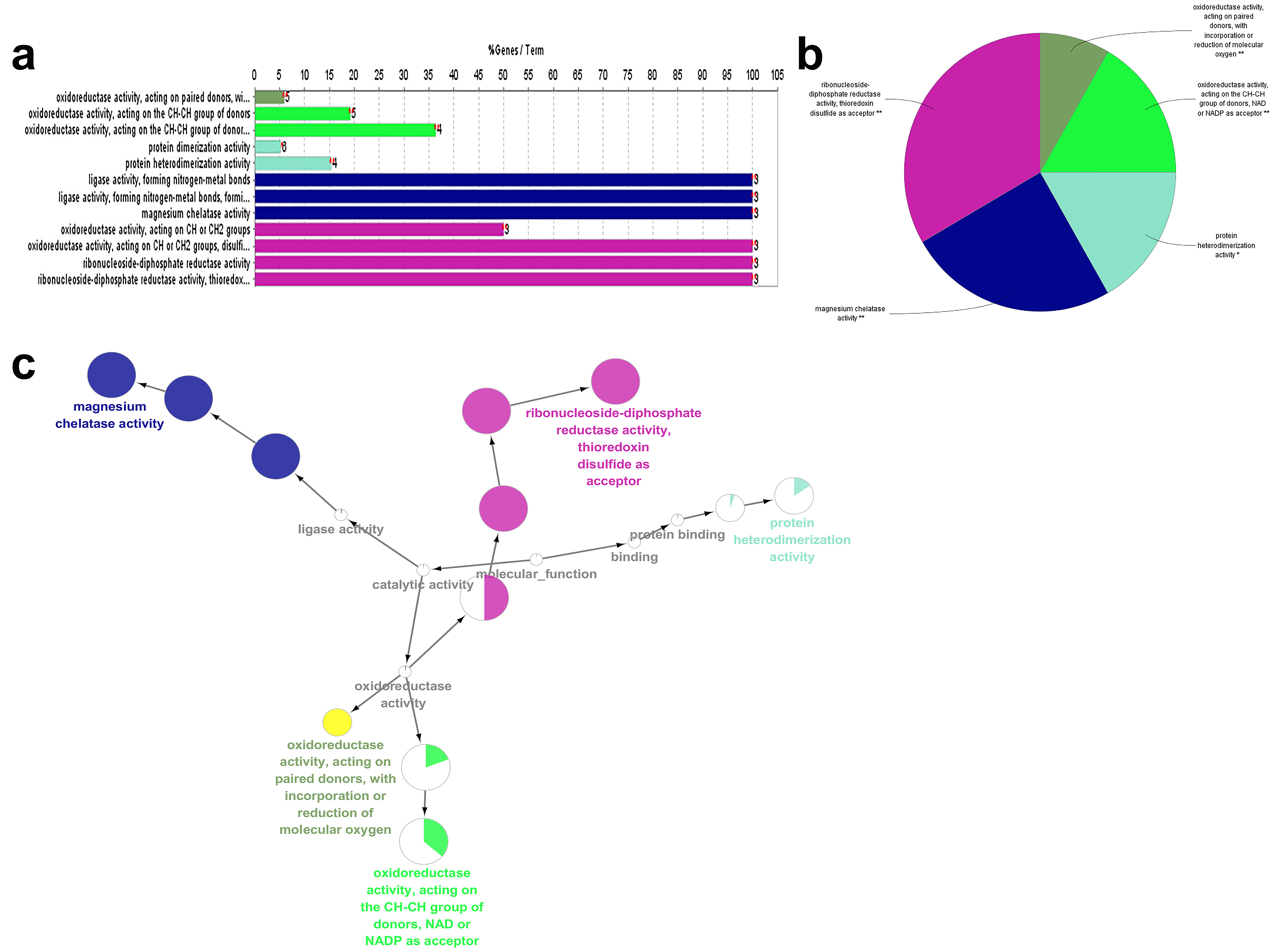


**Fig. S7 GO (molecular function) analysis of 152 known genes for CC and SG. (a)** GO terms specific for known genes. **(b)** Overview chart with functional groups including specific terms for known genes. **(c)** Functionally grouped network with terms as linked nodes based on their kappa score more than 0.3, where only the label of the most significant term per group is shown.


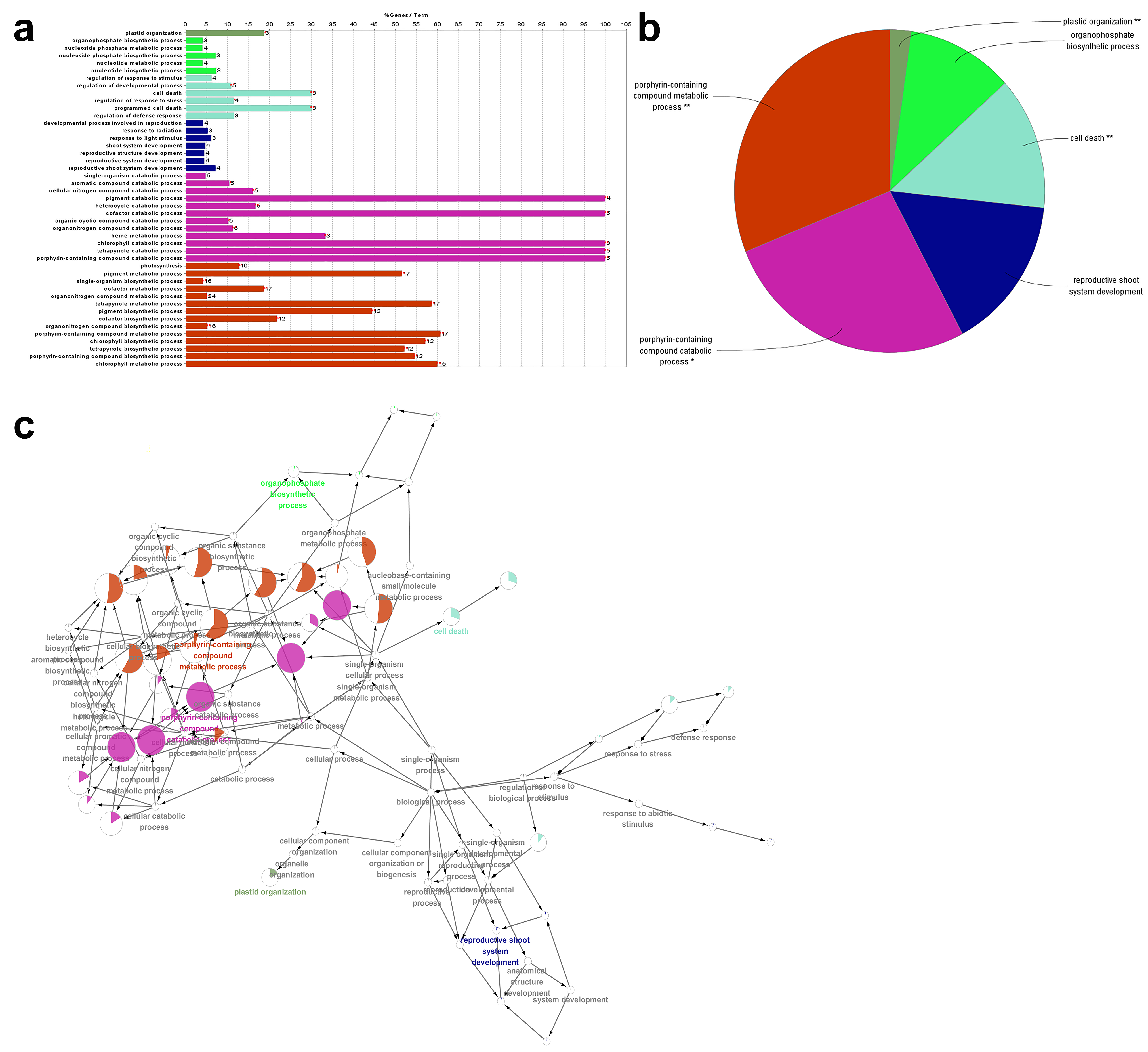


**Fig. S8 GO (biological process) analysis of 152 known genes for CC and SG. (a)** GO terms specific for known genes. **(b)** Overview chart with functional groups including specific terms for known genes. **(c)** Functionally grouped network with terms as linked nodes based on their kappa score more than 0.3, where only the label of the most significant term per group is shown.


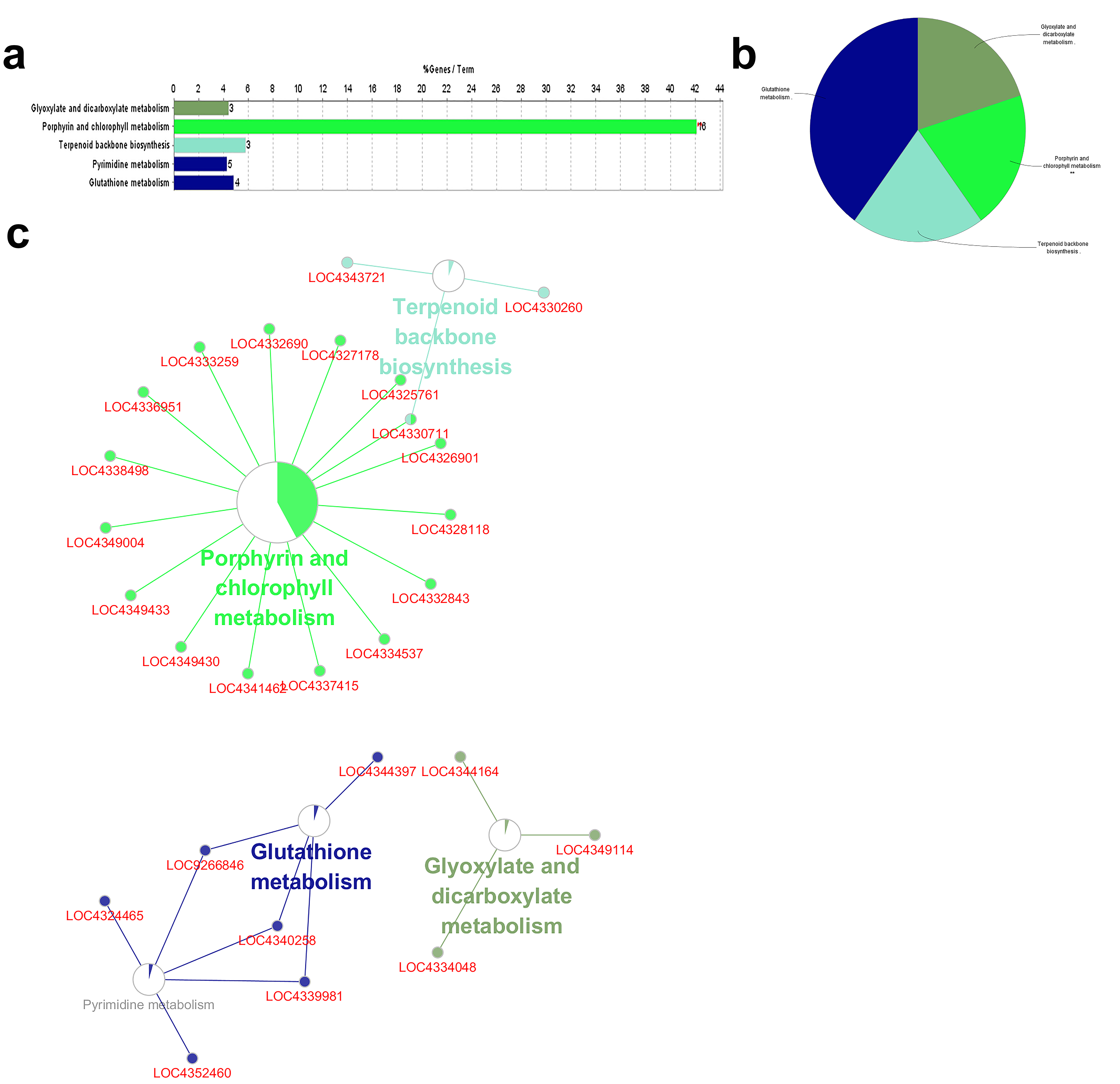


**Fig. S9 KEGG pathway analysis of 152 known genes for CC and SG. (a)** KEGG pathway terms specific for known genes. **(b)** Overview chart with functional groups including specific terms for known genes. **(c)** Network of known genes and KEGG pathway.

**Fig. S10 Allele analysis of four non-synonymous SNPs within *NOL* gene.** Comparisons of the four indices of rice CC and ACC between alleles at **(a)** Chr3_ 25519021, **(b)** Chr3_ 25523316, **(c)** Chr3_ 25525039 and **(d)** Chr3_ 25525141. Green violins show significantly higher SFH, TSH, CSF and TCS than yellow violins (*P* < 0.05).

**Fig. S11 Allele analysis of three non-synonymous SNPs within the *SSG4* gene.** Comparisons of the two CC indices between alleles at **(a)** Chr1_4133772, **(b)** Chr1_4134499, **(c)** Chr1_4134499. Green violins show significantly higher SFH and TSH than yellow violins (*P* < 0.05).

**Fig. S12 Allele analysis of eight non-synonymous SNPs within the *CHR729* gene.** Comparisons of the two CC indices of rice between alleles at **(a)** Chr7_18626131, **(b)** Chr7_18626400, **(c)** Chr7_18626503, **(d)** Chr7_18627606, **(e)** Chr7_18628210, **(f)** Chr7_18632361, **(g)** Chr7_18634846, **(h)** Chr7_18636157. In each subpopulation, green violins show significantly higher SFH and TSH than yellow violins (*P* < 0.05).

**Fig. S13 Allele analysis of two non-synonymous SNPs within the *OsFRDL1* gene.** Comparisons of the two ACC indices between alleles at **(a)** Chr3_ 6134135, **(b)** Chr3_6136209. Green violins show significantly higher CSF and TCS than yellow violins (*P* < 0.05).

**Fig. S14 Genome-wide association signals in the region 15-17 Mb on chromosome 7 for six indices.** Manhattan plots for SFH, TCH, ADSF, RDSF, CSF and TCS in **(a)** full population, **(b)** *indica* and **(c)** *japonica*. Red dots show all SNPs in candidate gene *OsSG1*.

**Fig. S15 LD heatmap of four lead SNPs associated with six indices on chromosome 7.**

**Fig. S16 Sequence alignment of *OsSG1* using three non-synonymous SNPs.**
